# Supplementary material for: Pyruvate Dehydrogenase Kinase 1 inhibition mediated oxidative phosphorylation enhancement in cartilage promotes osteoarthritis progression
Source: BMC Musculoskelet Disord. 2023 Jul 20;24:597. doi: 10.1186/s12891-023-06585-6 (PMC10357736; doi:10.1186/s12891-023-06585-6)
Supplement: Supplementary file 1 — Additional file 1. [file 12891_2023_6585_MOESM1_ESM.zip › BP.Gsea.1653623667859/GOBP_REGULATION_OF_FATTY_ACID_OXIDATION.html]

Details for gene set GOBP\_REGULATION\_OF\_FATTY\_ACID\_OXIDATION[GSEA]

|  || Dataset | 55457\_collapsed\_to\_symbols.cls.cls #OA\_versus\_normal.cls.cls #OA\_versus\_normal\_repos |
| Phenotype | cls.cls#OA\_versus\_normal\_repos |
| Upregulated in class | normal |
| GeneSet | GOBP\_REGULATION\_OF\_FATTY\_ACID\_OXIDATION |
| Enrichment Score (ES) | -0.53000444 |
| Normalized Enrichment Score (NES) | -1.7657852 |
| Nominal p-value | 0.011824325 |
| FDR q-value | 0.119070224 |
| FWER p-Value | 0.998 |
Table: GSEA Results Summary

  

Fig 1: Enrichment plot: GOBP\_REGULATION\_OF\_FATTY\_ACID\_OXIDATION      
 Profile of the Running ES Score & Positions of GeneSet Members on the Rank Ordered List

  

| SYMBOL | TITLE | RANK IN GENE LIST | RANK METRIC SCORE | RUNNING ES | CORE ENRICHMENT || 1 | APPL2 | "adaptor protein, phosphotyrosine interacting with PH domain and leucine zipper 2 [Source:HGNC Symbol;Acc:HGNC:18242]" | 586 | 0.628 | 0.0193 | No |
| 2 | AKT1 | AKT serine/threonine kinase 1 [Source:HGNC Symbol;Acc:HGNC:391] | 1192 | 0.478 | 0.0217 | No |
| 3 | SOX9 | SRY-box transcription factor 9 [Source:HGNC Symbol;Acc:HGNC:11204] | 2089 | 0.351 | -0.0117 | No |
| 4 | ABCD2 | ATP binding cassette subfamily D member 2 [Source:HGNC Symbol;Acc:HGNC:66] | 3031 | 0.246 | -0.0594 | No |
| 5 | PRKAG2 | protein kinase AMP-activated non-catalytic subunit gamma 2 [Source:HGNC Symbol;Acc:HGNC:9386] | 4362 | 0.123 | -0.1499 | No |
| 6 | SIRT4 | sirtuin 4 [Source:HGNC Symbol;Acc:HGNC:14932] | 4477 | 0.115 | -0.1469 | No |
| 7 | MLYCD | malonyl-CoA decarboxylase [Source:HGNC Symbol;Acc:HGNC:7150] | 4852 | 0.083 | -0.1674 | No |
| 8 | FABP3 | fatty acid binding protein 3 [Source:HGNC Symbol;Acc:HGNC:3557] | 5818 | 0.008 | -0.2416 | No |
| 9 | ACADVL | acyl-CoA dehydrogenase very long chain [Source:HGNC Symbol;Acc:HGNC:92] | 6394 | -0.034 | -0.2827 | No |
| 10 | ACADL | acyl-CoA dehydrogenase long chain [Source:HGNC Symbol;Acc:HGNC:88] | 6427 | -0.037 | -0.2813 | No |
| 11 | FABP1 | fatty acid binding protein 1 [Source:HGNC Symbol;Acc:HGNC:3555] | 7604 | -0.127 | -0.3595 | No |
| 12 | ABCB11 | ATP binding cassette subfamily B member 11 [Source:HGNC Symbol;Acc:HGNC:42] | 7825 | -0.143 | -0.3619 | No |
| 13 | CPT1A | carnitine palmitoyltransferase 1A [Source:HGNC Symbol;Acc:HGNC:2328] | 8046 | -0.159 | -0.3626 | No |
| 14 | ABCD1 | ATP binding cassette subfamily D member 1 [Source:HGNC Symbol;Acc:HGNC:61] | 8930 | -0.226 | -0.4078 | No |
| 15 | PPARGC1A | PPARG coactivator 1 alpha [Source:HGNC Symbol;Acc:HGNC:9237] | 10504 | -0.374 | -0.4914 | Yes |
| 16 | ACACB | acetyl-CoA carboxylase beta [Source:HGNC Symbol;Acc:HGNC:85] | 10955 | -0.425 | -0.4826 | Yes |
| 17 | PPARA | peroxisome proliferator activated receptor alpha [Source:HGNC Symbol;Acc:HGNC:9232] | 11110 | -0.445 | -0.4486 | Yes |
| 18 | CNR1 | cannabinoid receptor 1 [Source:HGNC Symbol;Acc:HGNC:2159] | 11181 | -0.454 | -0.4072 | Yes |
| 19 | PPARD | peroxisome proliferator activated receptor delta [Source:HGNC Symbol;Acc:HGNC:9235] | 11301 | -0.471 | -0.3678 | Yes |
| 20 | PDK4 | pyruvate dehydrogenase kinase 4 [Source:HGNC Symbol;Acc:HGNC:8812] | 11384 | -0.483 | -0.3243 | Yes |
| 21 | IRS2 | insulin receptor substrate 2 [Source:HGNC Symbol;Acc:HGNC:6126] | 11390 | -0.485 | -0.2747 | Yes |
| 22 | TWIST1 | twist family bHLH transcription factor 1 [Source:HGNC Symbol;Acc:HGNC:12428] | 11696 | -0.531 | -0.2435 | Yes |
| 23 | LONP2 | "lon peptidase 2, peroxisomal [Source:HGNC Symbol;Acc:HGNC:20598]" | 11699 | -0.532 | -0.1887 | Yes |
| 24 | IRS1 | insulin receptor substrate 1 [Source:HGNC Symbol;Acc:HGNC:6125] | 11841 | -0.557 | -0.1422 | Yes |
| 25 | NR4A3 | nuclear receptor subfamily 4 group A member 3 [Source:HGNC Symbol;Acc:HGNC:7982] | 12543 | -0.769 | -0.1172 | Yes |
| 26 | AKT2 | AKT serine/threonine kinase 2 [Source:HGNC Symbol;Acc:HGNC:392] | 12896 | -1.404 | 0.0003 | Yes |
Table: GSEA details [plain text format]

  

Fig 2: GOBP\_REGULATION\_OF\_FATTY\_ACID\_OXIDATION      
 Blue-Pink O' Gram in the Space of the Analyzed GeneSet

  

Fig 3: GOBP\_REGULATION\_OF\_FATTY\_ACID\_OXIDATION: Random ES distribution      
 Gene set null distribution of ES for **GOBP\_REGULATION\_OF\_FATTY\_ACID\_OXIDATION**
